# A Mediterranean-style eating pattern with lean, unprocessed red meat has cardiometabolic benefits for adults who are overweight or obese in a randomized, crossover, controlled feeding trial

**DOI:** 10.1093/ajcn/nqy075

**Published:** 2018-06-13

**Authors:** Lauren E O'Connor, Douglas Paddon-Jones, Amy J Wright, Wayne W Campbell

**Affiliations:** 1 Department of Nutrition Science, Purdue University, West Lafayette, IN; 2 Department of Nutrition and Metabolism, University of Texas Medical Branch, Galveston, TX

**Keywords:** beef, pork, healthy eating pattern, blood lipids, blood lipoproteins, blood pressure

## Abstract

**Background:**

A Mediterranean-style eating pattern (Mediterranean Pattern) is often described as being low in red meat. Research shows that lean, unprocessed red meat can be incorporated into healthy eating patterns to improve cardiometabolic disease (CMD) risk factors.

**Objective:**

We assessed the effects of consuming different amounts of lean, unprocessed red meat in a Mediterranean Pattern on CMD risk factors. We hypothesized that consuming a Mediterranean Pattern would improve CMD risk factors and that red meat intake would not influence these improvements.

**Design:**

In an investigator-blinded, randomized, crossover, controlled feeding trial, 41 subjects [mean ± SD age: 46 ± 2 y; mean ± SD body mass index (kg/m^2^): 30.5 ± 0.6] were provided with a Mediterranean Pattern for two 5-wk interventions separated by 4 wk of self-selected eating. The Mediterranean Patterns contained ∼500 g [typical US intake (Med-Red)] and ∼200 g [commonly recommended intake in heart-healthy eating patterns (Med-Control)] of lean, unprocessed beef or pork per week. Red meat intake was compensated by poultry and other protein-rich foods. Baseline and postintervention outcomes included fasting blood pressure, serum lipids, lipoproteins, glucose, insulin, and ambulatory blood pressure. The presented results were adjusted for age, sex, and body mass at each time point (*P* < 0.05).

**Results:**

Total cholesterol decreased, but greater reductions occurred with Med-Red than with Med-Control (−0.4 ± 0.1 and −0.2 ±0.1 mmol/L, respectively, intervention × time = 0.045]. Low-density lipoprotein decreased with Med-Red but was unchanged with Med-Control [−0.3 ± 0.1 and −0.1 ± 0.1 mmol/L, respectively, intervention × time = 0.038], whereas high-density lipoprotein (HDL) concentrations decreased nondifferentially [−0.1 ± 0.0 mmol/L]. Triglycerides, total cholesterol:HDL, glucose, and insulin did not change with either Med-Red or Med-Control. All blood pressure parameters improved, except during sleep, independent of the red meat intake amount.

**Conclusions:**

Adults who are overweight or moderately obese may improve multiple cardiometabolic disease risk factors by adopting a Mediterranean-style eating pattern with or without reductions in red meat intake when red meats are lean and unprocessed. This trial was registered at clinicaltrials.gov as NCT02573129.

## INTRODUCTION

The historically low chronic disease rates in Mediterranean countries are often attributed to eating habits. In the 1960s, a Mediterranean-style eating pattern (Mediterranean Pattern) was first recognized in a small cohort of coastal Greek olive farmers who had lower rates of cardiovascular disease than six other world regions (1). Their eating pattern was predominantly plant-based, notably low in red meat, and olive oil was the main source of fat (2). The health-promoting properties of a Mediterranean Pattern, including reduced risk of developing cardiovascular disease and type 2 diabetes, are supported by recent and larger studies (3–7). These recent studies, including the Prevención con Dieta Mediterránea (PREDIMED) (5) and Seguimiento Universidad de Navarra (SUN) cohorts (8), were largely conducted on Spaniards who had higher red meat intakes (∼700–1200 g/wk) (9) than the historic Greek olive farmers (∼245 g/wk) (10). These studies are mostly observational in nature and were not designed to directly compare consuming Mediterranean Patterns with different amounts of red meat intake on cardiometabolic disease risk factors (CMD).

Conclusions about the cardiometabolic risks of consuming red meat are historically inconsistent. The supporting literature base consists largely of observational cohort studies in which “red meat” is often ill-defined and grouped with processed meat as one intake category (11). This leads to inconsistent conclusions about the associations between red meat consumption and CMD (11). More recent observational research which assesses unprocessed red meat independently of processed meat shows little or no association between unprocessed red meat consumption and CMD (11, 12). In agreement, a compilation of randomized controlled trial data shows that total red meat, but mostly unprocessed beef and pork, consumption has no negative effect on cardiovascular disease risk factors (13). Nevertheless, US residents are encouraged to lower their red meat intake (14, 15).

The foundation for the recommendation to lower red meat intake in the context of a Mediterranean Pattern is unclear. US residents typically consume less red meat (11, 16) than what was reported in the large Mediterranean Pattern studies showing cardiometabolic benefits mentioned previously (5, 8). The primary objective of this controlled feeding trial was to assess the effects of consuming a Mediterranean Pattern with different amounts of red meat on CMD risk factors. We compared a Mediterranean Pattern with ∼500 g lean, unprocessed red meat/wk (Med-Red) and a Mediterranean Pattern with ∼200 g lean, unprocessed red meat/wk (Med-Control) because these are the amounts that are typically consumed by US residents (11, 16) and commonly recommended in heart-healthy eating patterns (17, 18), respectively. We hypothesized that the amount of red meat consumed would not influence Mediterranean Pattern-induced improvements in CMD risk factors of adults who are overweight or obese.

## METHODS

### Experimental design

This experimental design was a 16-wk randomized, crossover, investigator-blinded, controlled feeding study. Subjects consumed a Mediterranean Pattern for two 5-wk controlled feeding interventions separated by at least 4 wk of a self-selected and unrestricted eating pattern (washout). Dietary intake, body mass and composition, and CMD risk factors [including total cholesterol (total-C), LDL cholesterol, HDL cholesterol, total-C:HDL cholesterol, total apolipoprotein B (ApoB), triglycerides, glucose, insulin, HOMA-IR, C-reactive protein (CRP), fasting blood pressure, ambulatory blood pressure, and the Framingham Heart Study 10-y cardiovascular disease risk and vascular age] were measured at both baselines and during the last week of each Mediterranean Pattern intervention. Randomization was completed using an online randomization plan generator (http://www.randomization.com/). The trial was registered at clinicaltrials.gov as NCT02573129.

### Subjects

Subjects who were overweight or obese [BMI (kg/m^2^) 25–37], aged 30–69 y [representing middle-aged adults and adulthood life stage groups of the Dietary Reference Intakes (19)], and not already following a Mediterranean Pattern [as indicated by a score of <5 on the 14-item Mediterranean Diet Assessment Tool (20)] were recruited from the Greater Lafayette, IN area. Subject inclusion criteria were total-C <6.70 mmol/L, LDL cholesterol <4.10 mmol/L, triglycerides <4.5 mmol/L, fasting glucose <6.1 mmol/L, systolic blood pressure <160 mm Hg, diastolic blood pressure <100 mm Hg, body mass <140 kg, no acute illness, nonsmokers, normal liver and kidney functions, and non-diabetic. Subjects were required to be weight stable (±4.5 kg), to have consistent physical activity levels for 3 mo prior to starting the study, and to have stable medication use for 6 mo prior to and throughout the study. A physician reviewed each individual's screening measurements to ensure that they met the study inclusion criteria and to approve them for participation.

### Assessment of self-selected eating pattern

Before being randomized into the study, subjects completed the Mediterranean Diet Assessment Tool (20) to confirm that they were not already consuming a Mediterranean Pattern. Subjects were instructed to consume their self-selected unrestricted eating patterns (recorded with 3-d food logs) both during the baseline testing weeks and throughout the washout.

### Mediterranean Pattern

Menus were developed using Pronutra software (Viocare, Inc.) and followed the PREDIMED protocol (21) to achieve the desired Mediterranean Pattern. The menus were verified using the Mediterranean Diet Assessment Tool (20). Daily macronutrient intakes were targeted at 40% of total energy as carbohydrate, 22% protein, and 40% fat. Daily fat intakes were targeted at 7% of total energy as saturated fat and 20% monounsaturated fat. Med-Red and Med-Control differed predominantly in the amounts of red meat and poultry provided. Further adjustments were required to match the energy and macronutrients of the Med-Red and Med-Control menus, which was achieved by manipulation of mainly dairy, egg, and grain consumption. Fish and legume intake were similar in both Mediterranean Patterns in order to achieve the desired eating pattern per the PREDIMED protocol. Sodium, potassium, magnesium, and calcium intakes were targeted to be within ±15% between the Med-Red and Med-Control menus, and were calculated using the Linear Index Model (22). Each subject's energy requirement was estimated using sex-specific equations published by the Institute of Medicine (19), and menus were designed to maintain subjects’ baseline 1 body mass. Subjects were given the option to consume 150 mL of self-selected dry red wine daily.

All foods were prepared and provided to subjects during the two Mediterranean Pattern interventions by the NIH-supported Indiana Clinical Research Center Bionutrition Facility at Purdue University. The red meats and poultry provided were beef or pork tenderloins and chicken or turkey breasts (white meat with the skin removed prior to cooking). The meats were consumed in mixed heterogeneous dishes. All red meat and poultry provided was lean [<10 g total fat, <5 g saturated fat, and <95 mg cholesterol (23)]. All red meats and poultry underwent no further preservation processing beyond refrigeration or freezing (24), i.e., no smoking, curing, salting, or the addition of chemical preservatives (14). While meat processing terms vary, we use the term “unprocessed” throughout the article to be consistent with previous literature on this topic (11). Subjects weighed in and met with study staff weekly to monitor body mass and promote compliance, respectively. Subjects completed daily (and returned weekly) menu check-off lists to track self-reported deviations from the provided Mediterranean Pattern. Dietary intake and compliance were measured from the menu check-off lists of 3 d during the last week of each intervention.

### Body mass and composition

Body mass and composition (percentage body fat and fat-free mass) were measured at during both baselines periods and during the last week of each intervention via the BOD POD Gold Standard Body Composition Tracking System (COSMED USA, Inc.).

### Cardiometabolic disease risk factors

Cardiometabolic disease risk factors were measured for all subjects (*n* = 41) during both baseline periods and during the last week of each intervention. Fasting blood samples were collected from an antecubital vein into serum separator tubes and centrifuged for 15 min at 3.0 g and 4ºC. Fresh serum was then shipped to Mid America Clinical Laboratories to determine total-C, HDL cholesterol, triglycerides, and glucose concentrations via enzymatic colorimetry using oxidase methods on a COBAS Integra 400 Plus Analyzer (Roche Diagnostics Ltd). LDL cholesterol was calculated using the following equation: LDL cholesterol = total-C – [HDL cholesterol + (triglycerides/5)]. The remaining serum was divided into samples, stored at −80ºC, then thawed after all subjects had completed both interventions for analyses of insulin, total ApoB, and CRP concentrations. Fasting serum ApoB and CRP were measured via enzymatic colorimetry via oxidase methods on a COBAS Integra 400 Plus analyzer. Fasting serum insulin was measured via an electrochemiluminescence immunoassay on COBAS e411 analyzer (Roche Diagnostics Ltd).

Ambulatory and fasting blood pressures were measured during both baseline periods and during the last week of each intervention. Subjects wore an ambulatory blood pressure monitor for 48 h (Oscar2, Suntech Medical, Inc.). Blood pressure measurements were taken at 30 min intervals during the day (0800–2100) and at 90 min intervals through the night (2230–0730). Data were excluded from the analysis if >20% of scheduled measurements were invalid. Fasting blood pressures were measured in a quiet, dimly lit room. Measurements were taken after subjects sat upright for 15 min of rest (HEM-780, Omron Healthcare, Inc.). Two measurements were recorded (a third if the values differed by ≥3 mm Hg) and were averaged.

### Cardiometabolic disease risk prediction

Predictions of long-term cardiovascular disease risk and vascular age were calculated using the Framingham Heart Study 10-y cardiovascular disease risk lipid equation (25).

### Ethics

The study protocol and all study documents were approved by the Purdue University Biomedical Institutional Review Board (protocol #1501015662). All subjects provided written informed consent and received a monetary stipend.

### Statistics

Power calculations (G*Power version 3.1.9.2, Heinrich-Heine-Universität Düsseldorf) indicated that 40 subjects would provide >95% power to detect changes in fasting serum total-C and fasting systolic blood pressure, as achieved in a similar randomized crossover trial assessing the effects of consuming lean, unprocessed pork as opposed to chicken or fish in a Dietary Approaches to Stop Hypertension (DASH) eating pattern (α = 0.05) (26). We hypothesized that the inclusion of unprocessed red meat in a Mediterranean Pattern would not influence changes in these variables. Power calculation indicated that 40 subjects would provide >85% power to detect a differential response between Med-Red and Med-Control that was equal to half of the standard deviation of the response (effect size = 0.5).

All data were double entered independently and cross-checked for accuracy by the study manager (LEO). Data from 41 subjects who completed both interventions were analyzed via a doubly repeated-measures ANOVA using the PROC MIXED command in SAS version 9.4 (SAS Institute). This analysis measured: *1*) main effects of time (baseline compared with postintervention measurements; one-tailed), *2*) interaction of time and intervention (Med-Red changes compared with Med-Control changes; two-tailed), *3*) changes over time within Med-Red and within Med-Control (intervention-specific effect indicated by intervention × time *P* value < 0.05; one-tailed), *4*) comparison of Med-Red and Med-Control baseline measurements (intervention × time sliced by time; two-tailed), *5*) comparison of Med-Red and Med-Control preintervention measurements (intervention × time sliced by time; two-tailed), and *6*) comparison of baseline 1 and baseline 2 measurements (trial × time interaction sliced by time; two-tailed) to determine if subjects’ baseline 1 health status was re-established at baseline 2. These analyses were repeated using baseline and intervention alcoholic drink-equivalents per day as covariates. The PROC MIXED command in SAS uses maximum likelihood to account for missing data in dependent variables (27). The number of observations available at each time point for all outcome variables are listed in **Supplemental Tables 1** and **2**. All cardiometabolic outcomes of interest were controlled for age, sex, and body mass at each time point, and body mass and composition were controlled for age and sex. Results are presented as adjusted least squares (LS) means ± SEM, and *P* values are Tukey-Kramer adjusted for multiple comparisons (*P* < 0.05).

WWC has full access to all the data from this study and takes responsibility for its integrity and analysis. Summaries of LS means ± SEM (*n* = 41), raw means ± SD (*n* = 41), and sex-specific raw means ± SD for females and males are presented in **Supplemental Tables 1–4**, respectively. Primary deidentified data, analytical methods, and study materials are available upon request.

## RESULTS

### Subject characteristics

Fifty individuals were randomized into the study, but 18% (9) dropped out during week 1 of the first intervention. The remaining 41 subjects (28 women and 13 men) completed both interventions (see **Supplemental Figure 1**). Baseline 1 values of mean age, BMI, and fasting serum total-C, LDL cholesterol, HDL cholesterol, triglycerides, glucose, insulin concentrations, and fasting blood pressures are shown in **Table 1**.

**TABLE 1:** Subject characteristics at baseline 1^1^

Outcome	Baseline 1
Age, y	46 ± 2
BMI, kg/m^2^	30.5 ± 0.6
Total cholesterol, mmol/L	4.97 ± 0.13
LDL cholesterol, mmol/L	3.08 ± 0.10
HDL cholesterol, mmol/L	1.27 ± 0.05
Triglycerides, mmol/L	1.3 ± 0.1
Glucose, mmol/L	5.5 ± 0.1
Insulin, pmol/L	86.1 ± 8.3
Systolic/diastolic blood pressure, mm Hg	118 ± 2/80 ± 1
14-point Mediterranean Diet Assessment Tool (20)	4 ± 0

^1^Values are means ± SEMs. There were no differences between baseline 1 and baseline 2 measurements (*n* = 41). Conversion factors are available at: http://www.amamanualofstyle.com/page/si-conversion-calculator.

### Dietary intakes

Subjects were not consuming a Mediterranean Pattern at the start of the study, as indicated by a mean score of 4 ± 0 on the 14-item Mediterranean Diet Assessment tool (20). Self-reported dietary intake results from 3-d food logs did not differ between baseline 1 and baseline 2, confirming that subjects resumed their self-selected unrestricted eating patterns during the washout.

Mediterranean Diet Assessment Tool scores (20) increased ≥200%, as indicated by scores of 12 and 13 for the Med-Red and Med-Control menus, respectively. The Med-Red menu received one point less than Med-Control for the preferential use of red meat over poultry. The Med-Red and Med-Control menus had comparable daily energy contents, and intervention-specific macronutrient distributions were within ±1% (see **Table 2**). Daily or weekly servings of the US Dietary Guidelines for Americans designated food groups are shown in **Table 3** for representative Med-Red and Med-Control 7-d menu cycles. Mean self-reported compliance to the provided Med-Red and Med-Control menus were both ≥95%. Eleven subjects during Med-Red and 14 subjects during Med-Control consumed less than one 150-mL serving of wine/wk and were classified as non-wine drinkers. Among wine drinkers, 90 ± 3 mL of wine was consumed per day, on average, in both Med-Red (*n* = 15) and Med-Control (*n* = 12).

**TABLE 2:** Prescribed daily dietary intakes of the Mediterranean-style eating pattern menus^1^

	Med-Red	Med-Control
Energy, kcal	2601 ± 428	2573 ± 405^†^
Protein, %en	18 ± 0	19 ± 1^†^
Carbohydrate, %en	42 ± 1	42 ± 2
Fat, %en	40 ± 1	40 ± 1
Monounsaturated fat, %en	22 ± 1	21 ± 1^†^
Polyunsaturated fat, %en	8 ± 0	9 ± 1^†^
Saturated fat, %en	7 ± 0	8 ± 0^†^
Sodium, mg	2645 ± 354	2604 ± 317
Potassium, mg	4859 ± 624	4330 ± 653^†^
Magnesium, mg	490 ± 96	483 ± 74

^1^Intakes were averaged across a 7-d menu cycle. Results are presented as unadjusted means ± SDs (*n* = 41). ^†^Difference between Med-Red and Med-Control indicated by a paired *t*-test, *P* < 0.05. %en, percentage of total energy; Med-Control, Mediterranean-style eating pattern with ∼200 g lean, unprocessed red meat/wk; Med-Red, Mediterranean-style eating pattern with ∼500 g lean, unprocessed red meat/wk.

**TABLE 3:** Prescribed daily and weekly food group servings for the median energy intake level^1^

	Med-Red	Med-Control
Servings of fruit/d,^2^*n*	4	4
Servings of vegetables/d,^3^*n*	7	8
Dark green vegetables	1	2
Red and orange vegetables	1	1
Legumes	1	1
Starchy vegetables	1	1
Other vegetables	3	3
Servings of grains/d,^4^*n*	4	5
Whole grains	4	4
Refined grains	0	1
Protein-rich foods/wk,^5^ g		
Red meat	476	196
Poultry	112	420
Seafood	336	336
Whole eggs	2	3
Nuts, seed, soy^6^	560	616
Servings of dairy/d,^7^*n*	3	2
Olive oil/wk,^8^ g	247	247
14-point Mediterranean Diet Assessment Tool Score (20)	12	13

^1^Food group servings presented for representative 2492 kcal Med-Red and Med-Control diets averaged across a 7-d menu cycle. Med-Control, Mediterranean-style eating pattern with ∼200 g lean, unprocessed red meat/wk; Med-Red, Mediterranean-style eating pattern with ∼500 g lean, unprocessed red meat/wk.

^2^Half a cup or 1 medium fresh fruit.

^3^Half a cup of fresh or 1 cup of cooked vegetables.

^4^28 g = half a cup or 1 oz.

^5^28 g = 1 oz; cooked weights.

^6^28 g = 1 tbsp of nut butter, 0.5 oz of nuts or seeds, or ∼1 oz-equivalent.

^7^1 cup of milk or yogurt.

^8^4.5 g = 1 tsp.

### Body mass and composition

Chronologically, body mass at baseline 1 and baseline 2 did not differ. Body mass decreased more with Med-Red than Med-Control (−1.6 ± 0.5 vs. −1.0 ± 0.5 kg, intervention × time = 0.023), but postintervention values did not differ. Body fat percentage did not change with Med-Red or Med-Control.

### Cardiometabolic disease risk factors

**FIGURE 1:**
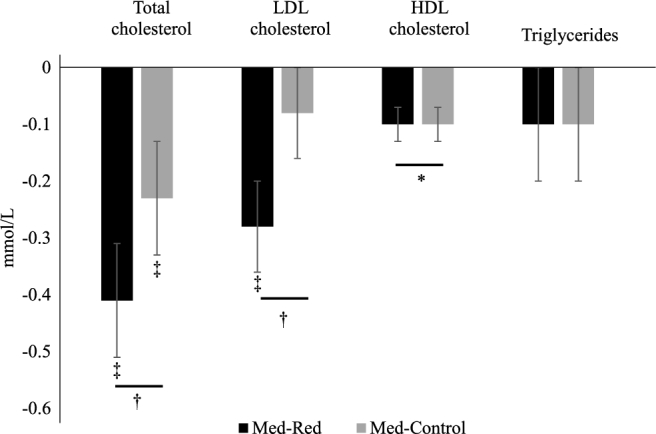
Changes in lipids and lipoproteins after consuming a Med-Red or Med-Control diet for 5 wk. Results are presented as LS means ± SEMs (*n* = 41). Data were analyzed using a doubly repeated-measures ANOVA adjusted for age, sex, and body mass at each time point. *Nondifferential change over time. ^†^Differential response between Med-Red and Med-Control when intervention × time *P* value < 0.05. ^‡^Intervention-specific change over time indicated by intervention × time *P* < 0.05. ApoB results followed a similar pattern as LDL cholesterol and are available in Supplemental Tables 1 and 2. Conversion factors are available at: http://www.amamanualofstyle.com/page/si-conversion-calculator. ApoB, apolipoprotein B; LS, least squares; Med-Control, Mediterranean-style eating pattern with ∼200 g lean, unprocessed red meat/wk; Med-Red, Mediterranean-style eating pattern with ∼500 g lean, unprocessed red meat/wk.

**FIGURE 2:**
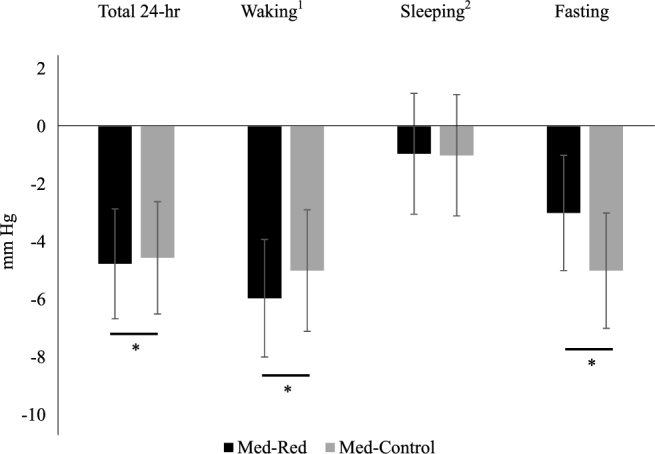
Changes in systolic blood pressures from consuming a Med-Red or Med-Control diet for 5 wk. Results are presented as LS means ± SEMs (*n* = 41). Data were analyzed using a doubly repeated-measures ANOVA adjusted for age, sex, and body mass at each time point. *Change over time. ^1^Waking blood pressure: 0800–2100. ^2^Sleeping blood pressure: 2230–0730. Diastolic blood pressure results followed similar patterns and are available in Supplemental Tables 1 and 2. LS, least squares; Med-Control, Mediterranean-style eating pattern with ∼200 g lean, unprocessed red meat/wk; Med-Red, Mediterranean-style eating pattern with ∼500 g lean, unprocessed red meat/wk.

Chronologically, measurements of CMD risk factors at baseline 1 and 2 did not differ. Med-Red decreased total-C 3% more than Med-Control. LDL cholesterol and ApoB decreased by 8% and 6%, respectively, with Med-Red, but did not change with Med-Control (see **Figure 1**). Total-C:HDL cholesterol, triglycerides, CRP, glucose, insulin, and HOMA-IR score did not change with Med-Red or Med-Control. Fasting and ambulatory blood pressure parameters improved with both Mediterranean Patterns, except during sleep, independent of red meat intake amount (see **Figure 2**). There were no differences between postintervention values of Med-Red and Med-Control for any CMD risk factors. Our results showed no difference between males and females in Mediterranean Pattern-induced cardiometabolic changes, independent of red meat intake amount. When considering baseline and intervention drink-equivalents as a covariate, there were still greater reductions in total-C with Med-Red, and reductions in LDL cholesterol with Med-Red but no changes with Med-Control, but the overall time effect and intervention-specific effects on ApoB diminished. Adjusted means ± SEMs and unadjusted means ± SDs for all CMD risk factors are available in Supplemental Tables 1 and 2, respectively. Sex-specific unadjusted means ± SDs are available in Supplemental Tables 3 and 4.

### Cardiovascular disease risk prediction

Framingham Heart Study 10-y cardiovascular disease risk decreased by 1% and vascular age increased by 2–3 y with a Mediterranean Pattern, independent of red meat intake amount.

## DISCUSSION

Simultaneously adopting a Mediterranean Pattern and reducing red meat intake is commonly recommended to decrease CMD risk (14, 15). Our results show that adopting a Mediterranean Pattern with or without reducing red meat intake improves CMD risk factors if the red meat is lean and unprocessed. Our results support previous findings that consuming lean, unprocessed red meat [∼120 g pork (26), ≤153 g beef (28–30), or ∼86 g lean beef, veal, or lamb (31)/d] does not hinder the effectiveness of a DASH pattern to improve CMD risk factors in the absence of clinically meaningful body mass reductions.

The American Heart Association and the American College of Cardiology declare inconsistent effects of consuming a Mediterranean Pattern on blood lipid and lipoprotein concentrations (32). The randomized controlled trials referenced by these societies are largely dietary counseling interventions and have inadequate control groups (33–35). Our study provided a novel opportunity to assess the effects of a Mediterranean Pattern in a tightly controlled crossover trial. Adopting a Mediterranean Pattern improved overall CMD risk factor profiles. However, reductions in LDL cholesterol and ApoB concentrations were largely attributable to Med-Red because there were no changes in these outcomes with Med-Control. Our results indicate that variations in Mediterranean Pattern compositions (36), such as meat source, may help explain inconsistent effects described by the American Heart Association and the American College of Cardiology (32). Further, meat source in our study did not affect Mediterranean Pattern-induced improvements in predictions of long-term cardiovascular disease risk (Framingham Heart Study 10-y cardiovascular disease risk and vascular age). These results are consistent with evidence that a Mediterranean Pattern decreases the risk of coronary heart disease, stroke, and total mortality (37), but changes in atherosclerosis-promoting lipid and lipoprotein concentrations may not be the mechanism (38, 39).

This study was not designed to identify mechanisms by which lean, unprocessed red meat consumption might differentially affect atherosclerosis-promoting lipids and lipoprotein concentrations. One speculation is that the greater body mass loss with Med-Red may be a mediating factor. Despite randomization of trial order, the baseline Med-Red body mass was quantitatively, but not statistically, 0.7 kg higher than the baseline Med-Control body mass. It is perhaps noteworthy that participants lost 0.6 kg more during Med-Red than during Med-Control, which was a statistically significant difference. Both of these body mass changes were modest (Med-Red: −1.8%; Med-Control: −1.1%), body masses were not different at the end of the interventions, and there were no differential changes in absolute or relative fat or fat-free masses. We controlled for body weight at each time point in our statistical model, and body mass was not a significant covariate for total-C (*P* = 0.321) or LDL cholesterol (*P* = 0.125), but was for ApoB (*P* = 0.035). The combination of the small magnitude of difference between Med-Red and Med-Control body mass changes (clinical relevancy of 0.6 kg difference) and the lack of significance in our statistical model suggests that the differential effects in total-C, LDL cholesterol, and ApoB are not because of differences in body mass. However, an impact of changes in body mass on changes in LDL cholesterol cannot be ruled out.

Meta-analyses of prospective cohort studies show that each 100-g serving unprocessed red meat/d increases the risk of developing type 2 diabetes by 19% (11, 40), but there is a paucity of experimental evidence to support this. Our Mediterranean Pattern study and the weight maintenance DASH Pattern studies previously mentioned (26, 29) showed no effect of these eating patterns on fasting glucose, insulin, or HOMA-IR, independent of red meat intake. One study compared the effects of energy-restricted DASH Patterns substituting plant protein with beef (12, 139, or 196 g lean unprocessed beef) combined with exercise on metabolic syndrome outcomes. The researchers concluded that weight loss was the primary modifier of metabolic improvements, independent of protein source (30). These studies support that Med and DASH Patterns are typically not effective at improving metabolic markers in the absence of weight loss or exercise (41–44). These eating patterns, particularly over the short term, are not suitable to assess the effects of red meat intake on changes in glycemic control. Future randomized controlled trials are warranted to assess the effects of lean, unprocessed red meat consumption on type 2 diabetes risk factors in eating patterns known to improve these outcomes.

There are different ways of quantifying the effectiveness of a nutrition intervention on CMD outcomes. Most commonly, researchers compare changes between groups or the differences between groups at the end of each intervention. In our study, 40 subjects provided >95% power to detect changes in fasting serum total-C and systolic blood pressure, and >85% power to detect a differential response between Med-Red and Med-Control. It is noteworthy that the postintervention values did not differ between Med-Red and Med-Control for any of the CMD risk factors measured, including those that showed differential changes (total-C, LDL cholesterol, and ApoB). The end of intervention values show that meat source did not influence Mediterranean Pattern–induced cardiometabolic responses. These results are consistent with previous studies that showed no postintervention differences in CMD risk factors between traditional DASH Patterns and DASH Patterns with higher red meat intake and similar macronutrient distributions (26, 28, 29).

Our randomized controlled trial is strengthened by a low drop-out rate (<18%) and a successful washout period (baseline 1 measures were re-established at baseline 2), but is not without limitations. The self-reported >95% menu compliance was not objectively confirmed. Our results are not generalizable to all cuts of beef and pork because only tenderloins were provided to subjects. Future studies should include various types of lean, unprocessed red meat in a feasibility study to follow up on our findings. We were unable to supply or encourage consumption of red wine owing to university regulations, but slight differences in wine intake between the Med-Red and Med-Control groups did not influence the results. Although unintentional, 98% of our sample population was Caucasian. Future research is needed to assess whether race and/or ethnicity influences responses.

The 2000-kcal Mediterranean Pattern proposed by the Dietary Guidelines Advisory Committee (DGAC) contains ∼300 g red meat/wk (45). The supporting literature base is largely prospective cohort studies that assess associations between red meat consumption and chronic disease in the context of a Western-style eating pattern (40, 46–48). Unhealthy lifestyle behaviors are correlated with red meat intake in this population which confounds the positive associations between red meat and chronic disease risk (49). The Mediterranean Pattern studies identified by the DGAC show low chronic disease risk with red and processed meat consumption up to ∼1200 g/wk for a 2000-kcal diet (9). Our results, as well as the Mediterranean Pattern studies identified in the report, do not support red meat reductions in the context of a Mediterranean Pattern. Further, the DGAC did not assess the health effects of unprocessed red meat independent of processed meats (which includes red meat and poultry). There is building evidence that unprocessed red meat consumption has little to no influence on cardiometabolic disease risk compared with processed meats (11, 12). Future DGACs need not only to consider the amount of red meat included in a Mediterranean Pattern, but also to be cognizant of the leanness and degree of meat processing.

In conclusion, adults who are overweight or obese can consume typical US intake quantities of red meat (∼70 g/d) as lean and unprocessed beef and pork when adopting a Mediterranean Pattern to improve cardiometabolic disease risk factors. Our results support previous observational and experimental evidence which shows that unprocessed and/or lean red meat consumption does not increase the risk of developing cardiovascular disease (11) or impair associated risk factors (13).

## Supplementary Material

nqy075_Supplement_Files

## Notes

This study was funded in part by the Beef Checkoff, the Pork Checkoff, the National Institute of Health's Ingestive Behavior Research Center at Purdue University (5T32DK076540-08), and the National Institute of Health's Indiana Clinical and Translational Sciences Institute. These organizations had no role in the design or conduct of the study; collection, analysis, or interpretation of the data; or writing of the manuscript.

Supplemental Tables 1–4 and Supplemental Figure 1 are available from the “Supplementary data” link in the online posting of the article and from the same link in the online table of contents at https://academic.oup.com/ajcn/.

Abbreviations used:

ApoB: apolipoprotein B
CMD: cardiometabolic disease
CRP: C-reactive protein
DASH: Dietary Approaches to Stop Hypertension
DGAC: Dietary Guidelines Advisory Committee
LS: least squares
Med-Control: Mediterranean-style eating pattern with ∼200 g of lean, unprocessed red meat per week
Mediterranean Pattern: Mediterranean-style eating pattern
Med-Red: Mediterranean-style eating pattern with ∼500 g of lean, unprocessed red meat per week
PREDIMED: Prevención con Dieta Mediterránea
total-C: total cholesterol
