# Supplementary material for: A Mediterranean-style eating pattern with lean, unprocessed red meat has cardiometabolic benefits for adults who are overweight or obese in a randomized, crossover, controlled feeding trial
Source: Am J Clin Nutr. 2018 Jun 13;108(1):33–40. doi: 10.1093/ajcn/nqy075 (PMC6600057; doi:10.1093/ajcn/nqy075)
Supplement: nqy075_Supplement_Files [file nqy075_supplement_files.pdf]

Supplemental Figure 1: Study recruitment flow diagram

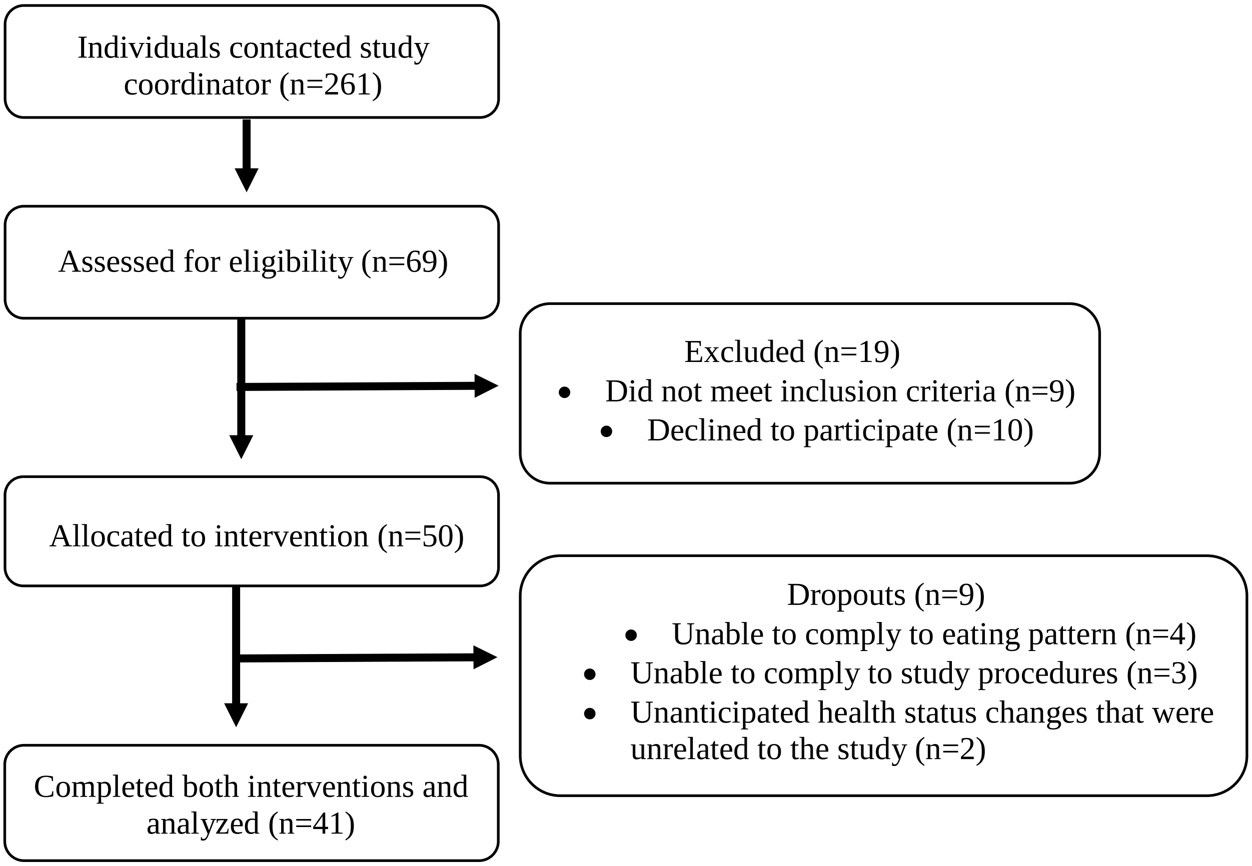

Supplemental Table 1: Cardiometabolic responses from consuming Med-Red vs Med-Control for 5 weeks

	Med-Red						Med-Control						P values	
	Baseline		Post		Change		Baseline		Post		Change			
Outcome	mean ± SEM	n	mean ± SEM	n	mean ± SEM	n	mean ± SEM	n	mean ± SEM	n	mean ± SEM	n	Time	Time* intervention
Body mass (kg)	91.2 ± 1.5	41	89.7 ± 1.5	40	-1.6 ± 0.5‡	40	90.6 ± 1.5	41	89.6 ± 1.5	41	-1.0 ± 0.5‡	41	0.006	0.023
Body fat (%)	37.2 ± 1.0	41	36.7 ± 1.0	40	-0.5 ± 0.5	40	36.6 ± 1.0	41	36.0 ± 1.0	41	-0.6 ± 0.5	41	0.117	0.738
Total cholesterol (mmol/L)	4.9 ± 0.1	41	4.5 ± 0.1	41	-0.4 ± 0.1‡	41	4.9 ± 0.1	41	4.7 ± 0.1	41	-0.2 ± 0.1‡	41	0.001	0.045
Low-density lipoprotein cholesterol (mmol/L)	3.1 ± 0.1	41	2.8 ± 0.1	41	-0.3 ± 0.1‡	41	3.0 ± 0.1	41	3.0 ± 0.1	41	-0.1 ± 0.1	41	0.016	0.038
High-density lipoprotein cholesterol (mmol/L)	1.2 ± 0.1	41	1.1 ± 0.0	41	-0.1 ± 0.0	41	1.2 ± 0.1	41	1.1 ± 0.0	41	-0.1 ± 0.0	41	0.002	0.622
Total cholesterol: high-density lipoprotein cholesterol	4.2 ± 0.1	41	4.2 ± 0.1	41	0.0 ± 0.1	41	4.1 ± 0.1	41	4.3 ± 0.1	41	0.1 ± 0.1	41	0.348	0.113
Apolipoprotein B (g/L)	1.0 ± 0.0	41	0.9 ± 0.0	41	-0.1 ± 0.0‡	41	0.9 ± 0.0	41	0.9 ± 0.0	40	0.0 ± 0.0	40	0.052	0.041
Triglycerides (mmol/L)	1.4 ± 0.1	41	1.3 ± 0.1	41	-0.1 ± 0.1	41	1.3 ± 0.1	41	1.2 ± 0.1	41	-0.1 ± 0.1	41	0.079	0.579
Glucose (mmol/L)	5.4 ± 0.1	41	5.3 ± 0.1	41	-0.1 ± 0.1	41	5.3 ± 0.1	41	5.3 ± 0.1	41	-0.1 ± 0.1	41	0.079	0.827
Insulin (pmol/L)	85.4 ± 7.6	41	75.0 ± 6.9	41	-9.7 ± 6.9	41	77.1 ± 6.9	41	70.8 ± 6.9	40	-9.7 ± 6.9	40	0.103	0.527
HOMA-IR	2.981 ± 0.299	41	2.557 ± 0.275	41	-0.445 ± 0.273	41	2.679 ± 0.297	41	2.432 ± 0.275	40	-0.247 ± 0.274	40	0.077	0.551
C-reactive protein (nmol/L)	21.9 ± 2.9	41	21.0 ± 2.9	41	-1.0 ± 2.9	41	21.9 ± 2.9	41	22.9 ± 2.9	40	0.1 ± 2.9	40	0.497	0.496
Total 24-hr SBP (mm Hg)	130 ± 2	39	126 ± 2	36	-5 ± 2	35	131 ± 2	37	126 ± 2	33	-5 ± 2	32	0.002	0.829
Waking SBP[1] (mm Hg)[2]	135 ± 2	40	129 ± 2	36	-6 ± 2	36	134 ± 2	37	129 ± 2	34	-5 ± 2	33	0.002	0.511
Sleeping SBP (mm Hg)[3]	115 ± 2	38	114 ± 2	37	-1 ± 2	36	119 ± 2	39	118 ± 2	35	-1 ± 2	34	0.203	0.874
Fasting SBP (mm Hg)	118 ± 2	41	114 ± 1	41	-3 ± 2	41	120 ± 2	41	115 ± 1	41	-5 ± 2	41	0.002	0.261
Total 24-hr DBP (mm Hg)	79 ± 1	39	75 ± 1	36	-4 ± 1	35	78 ± 1	37	75 ± 1	33	-2 ± 1	32	0.001	0.155
Waking DBP[4] (mm Hg) [1]	82 ± 1	40	77 ± 1	36	-5 ± 1	36	80 ± 1	37	77 ± 1	34	-3 ± 2	33	0.001	0.098
Sleeping DBP (mm Hg) [2]	67 ± 1	38	66 ± 1	37	-1 ± 1	36	69 ± 1	39	68 ± 1	35	-1 ± 1	34	0.162	0.724
Fasting DBP (mm Hg)	80 ± 1	41	77 ± 1	41	-3 ± 1	41	80 ± 1	41	78 ± 1	41	-2 ± 1	41	0.004	0.693
10-year risk (%)	4.6 ± 0.5	41	4.0 ± 0.4	41	-0.7 ± 0.4	41	4.6 ± 0.5	41	4.1 ± 0.4	41	-0.5 ± 0.4	41	0.047	0.611

Vascular age (years)	45 ± 2	41	42 ± 2	41	-3 ± 1.2	41	45 ± 2	41	42 ± 2	41	-2 ± 1.2	41	0.018	0.216

Data are presented as LS means ± SEM. Data were analyzed using a doubly repeated-measures ANOVA adjusted for age, sex, and body mass at each time point. [‡]indicates a change over time within Pattern from post-hoc analysis of intervention*time $p<0.05$. [1]SBP: systolic blood pressure, [2]waking blood pressure: 0800-2100, [3]sleeping blood pressure: 2230-0730, and [4]DBP: diastolic blood pressure. Conversion factors are available at: http://www.amamanualofstyle.com/page/si-conversion-calculator. Med-Control, Mediterranean-style eating pattern with ~200 g of lean unprocessed red meat; Med-Red, Mediterranean-style eating pattern with ~500 g of lean unprocessed red meat.

Supplemental Table 2: Unadjusted means, SD, and n at each time point of Med-Red and Med-Control

	**Med-Red**									**Med-Control**								
	**Baseline**			**Post**			**Change**			**Baseline**			**Post**			**Change**		
Outcome	mean	SD	n	mean	SD	n	mean	SD	n	mean	SD	n	mean	SD	n	mean	SD	n
Body mass (kg)	87.5	16	41	86.2	15.4	40	-1.7	1.7	40	86.8	16.1	41	85.8	15.4	41	-1.0	1.7	41
Body fat (%)	39.1	8.8	41	38.5	8.4	40	-0.5	1.7	40	38.5	9.4	41	37.9	9.2	41	-0.7	2.6	41
Total cholesterol (mmol/L)	5.00	0.88	41	4.56	0.70	41	-0.44	0.60	41	4.97	0.80	41	4.71	0.93	41	-0.23	0.49	41
LDL cholesterol (mmol/L)	3.11	0.75	41	2.80	0.57	41	-0.31	0.41	41	3.06	0.65	41	2.98	0.75	41	-0.10	0.49	41
HDL cholesterol (mmol/L)	1.27	0.34	41	1.19	0.31	41	-0.08	0.21	41	1.30	0.36	41	1.19	0.31	41	-0.10	0.13	41
Total cholesterol: high-density lipoprotein cholesterol	4.21	1.21	41	4.08	1.15	41	-0.12	0.53	41	4.12	1.21	41	4.20	1.18	41	0.08	0.59	41
Total apolipoprotein B (g/L)	1.0	0.2	41	0.9	0.2	41	-0.1	0.1	41	0.9	0.2	41	0.9	0.2	40	0.0	0.1	40
Triglycerides (mmol/L)	1.4	0.8	41	1.3	0.6	41	-0.1	0.5	41	1.4	0.7	41	1.3	0.6	41	-0.1	0.5	41
Glucose (mmol/L)	5.4	0.5	41	5.3	0.4	41	-0.1	0.4	41	5.4	0.5	41	5.3	0.4	41	-0.1	0.4	41
Insulin (pmol/L)	92	58	41	78	54	41	-13	44	41	83	51	41	75	45	40	-8	29	40
HOMA-IR	3.253	2.121	41	2.715	2.039	41	-0.538	1.636	41	2.924	2.004	41	2.602	1.668	40	-0.346	1.182	40
C-reactive protein (nmol/L)	22.95	17.52	41	20.95	19.81	41	-2.10	15.91	41	22.95	17.62	41	23.81	21.14	40	0.48	16.19	40
Total 24-hr SBP (mm Hg)	131	11	39	126	10	36	-5	11	35	131	12	37	125	10	33	-4	10	32
Waking SBP[1] (mm Hg)[2]	135	12	40	129	11	36	-7	12	36	134	12	37	128	11	34	-5	11	33
Sleeping SBP (mm Hg)[3]	116	10	38	115	12	37	-1	11	36	120	14	39	118	11	35	-2	14	34
Fasting SBP (mm Hg)	116	12	41	113	10	41	-3	9	41	118	13	41	113	10	41	-5	10	41
Total 24-hr DBP[4] (mm Hg)	78	7	39	74	7	36	-4	6	35	78	7	37	75	6	33	-2	6	32
Waking DBP (mm Hg)[1]	81	8	40	77	8	36	-6	7	36	80	7	37	76	8	34	-3	8	33
Sleeping DBP (mm Hg)[2]	67	7	38	66	7	37	-1	6	36	68	8	39	68	7	35	-1	7	34
Fasting DBP (mm Hg)	80	8	41	77	6	41	-3	6	41	80	8	41	78	7	41	-2	7	41
10-year risk (%)	5	4	41	4	3	41	-0.7	1.5	41	5	4	41	4	3	41	-0.5	2.0	41
Vascular age (years)	45	13	41	42	11	41	-2.7	5.5	41	45	14	41	43	12	41	-2.1	5.4	41

[1]SBP: systolic blood pressure, [2]waking blood pressure: 0800-2100, [3]sleeping blood pressure: 2230-0730, and [4]DBP: diastolic blood pressure. Conversion factors are available at: http://www.amamanualofstyle.com/page/si-conversion-calculator. Med-Control, Mediterranean-style eating pattern with ~200 g of lean unprocessed red meat; Med-Red, Mediterranean-style eating pattern with ~500 g of lean unprocessed red meat.

Supplemental Table 3: Raw mean ± SD at each time point of Med-Red and Med-Control for females (n=28, 48±10 yrs)

	**Med-Red**				**Med-Control**			
	**Baseline**		**Post**		**Baseline**		**Post**	
Outcome	mean	SD	mean	SD	mean	SD	mean	SD
Body mass (kg)	80.4	8.5	79.2	8.2	79.7	8.6	79.0	8.2
Body fat (%)	42.8	5.6	41.8	5.6	41.7	7.1	41.5	6.4
Total cholesterol (mmol/L)	5.01	0.89	4.55	0.75	5.04	0.84	4.85	0.98
LDL cholesterol (mmol/L)	3.05	0.78	2.72	0.60	3.03	0.66	2.99	0.77
HDL cholesterol (mmol/L)	1.34	0.29	1.27	0.33	1.42	0.37	1.27	0.32
Total cholesterol: high-density lipoprotein cholesterol	3.87	1.00	3.74	1.02	3.72	0.97	3.97	1.03
Total apolipoprotein B (g/L)	0.9	0.2	0.9	0.2	0.9	0.2	0.9	0.2
Triglycerides (mmol/L)	1.3	0.7	1.2	0.5	1.3	0.7	1.3	0.6
Glucose (mmol/L)	5.4	0.4	5.4	0.4	5.5	0.5	5.3	0.5
Insulin (pmol/L)	84.4	47.6	76.1	54.0	82.1	50.9	70.9	40.1
HOMA-IR	2.994	1.787	2.683	2.067	2.945	2.124	2.465	1.494
C-reactive protein (nmol/L)	20.9	15.3	21.9	21.4	23.4	18.6	22.6	18.0
Total 24-hr SBP (mm Hg)	130	10	125	10	130	12	124	10
Waking SBP[1] (mm Hg)[2]	134	11	129	12	133	11	126	11
Sleeping SBP (mm Hg)[3]	115	11	114	11	118	14	119	13
Fasting SBP (mm Hg)	113	9	110	9	116	12	110	10
Total 24-hr DBP[4] (mm Hg)	77	7	73	6	77	7	74	6
Waking DBP (mm Hg)[1]	80	7	75	8	79	7	75	8
Sleeping DBP (mm Hg)[2]	65	7	65	7	67	8	67	6
Fasting DBP (mm Hg)	79	7	77	7	80	8	78	7
10-year risk (%)	3.6	2.6	3.3	2.2	3.7	2.5	3.2	2.1
Vascular age (years)	44	14	43	13	45	14	42	13

Supplemental Table 4: Raw mean ± SD at each time point of Med-Red and Med-Control for males (n=13, 42±10 yrs)

	**Med-Red**				**Med-Control**			
	**Baseline**		**Post**		**Baseline**		**Post**	
Outcome	mean	SD	mean	SD	mean	SD	mean	SD
Body mass (kg)	102.7	17.8	100.7	17.1	102.2	17.9	100.5	17.2
Body fat (%)	31.1	9.1	31.2	9.0	31.7	10.2	30.0	9.5
Total cholesterol (mmol/L)	5.00	0.91	4.58	0.62	4.82	0.70	4.46	0.79
LDL cholesterol (mmol/L)	3.19	0.70	2.93	0.51	3.14	0.62	2.93	0.76
HDL cholesterol (mmol/L)	1.08	0.35	0.98	0.18	1.00	0.16	0.98	0.19
Total cholesterol: high-density lipoprotein cholesterol	4.92	1.35	4.81	1.11	4.97	1.25	4.69	1.38
Total apolipoprotein B (g/L)	1.0	0.2	0.9	0.2	1.0	0.2	0.9	0.3
Triglycerides (mmol/L)	1.6	0.9	1.4	0.8	1.5	0.7	1.2	0.5
Glucose (mmol/L)	5.4	0.6	5.2	0.5	5.3	0.5	5.3	0.5
Insulin (pmol/L)	107.3	76.5	83.8	58.7	83.8	52.6	83.6	54.9
HOMA-IR	3.812	2.752	2.783	2.104	2.877	1.798	2.886	2.017
C-reactive protein (nmol/L)	27.4	21	18.9	16.7	22.2	16.2	26.4	27.2
Total 24-hr SBP (mm Hg)	134	11	128	11	133	12	127	10
Waking SBP[1] (mm Hg)[2]	138	14	131	11	136	13	130	12
Sleeping SBP (mm Hg)[3]	119	9	118	13	124	12	118	8
Fasting SBP (mm Hg)	124	13	120	9	124	13	121	6
Total 24-hr DBP[4] (mm Hg)	80	9	77	7	79	7	77	7
Waking DBP (mm Hg)[1]	83	10	80	7	81	8	79	8
Sleeping DBP (mm Hg)[2]	69	7	67	7	70	8	70	7
Fasting DBP (mm Hg)	82	9	79	6	80	9	77	5
10-year risk (%)	6.8	4.8	6.0	3.5	6.1	4.9	5.8	3.7
Vascular age (years)	45	12	44	10	44	11	43	10
